# Validated TLC-densitometry method for the simultaneous analysis of pyrethroid insecticides in agricultural and domestic products

**DOI:** 10.1186/1752-153X-6-93

**Published:** 2012-08-31

**Authors:** Syed Ghulam Musharraf, Muhammad Shoaib, Dileep Kumar, Muhammad Najam-ul-Haq

**Affiliations:** 1 H.E.J. Research Institute of Chemistry, International Center for Chemical and Biological Sciences, University of Karachi, Karachi, 75270, Pakistan; 2 Division of Analytical Chemistry, Institute of Chemical Sciences, Bahauddin Zakariya University (B.Z.U.), Multan, 60800, Pakistan

**Keywords:** Pyrethroid insecticides, Agricultural and domestic products, TLC-densitometry

## Abstract

**Background:**

Pyrethroids are widely used for the control of pests and insects, as pyrethroids are believed to pose little risk to human health and environment. However, exposure to the pyrethroids exceeding the label directions might adversely affect human health and environment. Hence a careful selection of environment friendly household product is required that must contain exactly the label claimed pyrethroids amount.

**Results:**

A sensitive and robust TLC-densitometric method for simultaneous quantification of commonly used synthetic pyrethroids including esbiothrin, alpha-cypermethrin and *cis*/*trans* permethrin in agricultural and domestic products has been developed and validated. TLC aluminum sheets, precoated with 0.2 mm thick layer of silica gel 60 F-254, were used for chromatographic process. Densitometric analysis of chromatoplates was carried out in absorbance mode at corresponding λ_max_ of each pyrethroid. Equally valid common mobile phase for all pyrethroids consisted of hexane-dichloromethane-ethylacetate-formic acid (8:1.5:0.4:0.1 v/v/v/v) which provided sharp and symmetrical peaks of esbiothrin, alpha-cypermethrin, *trans*-permethrin and *cis*-permethrin, at *R*_f_ 0.31, 0.53, 0.6 and 0.65, respectively. Linear regression data for respective calibration curves showed a good linearity for all pyrethroids with *r* = 0.991-0.996. Limits of detection (LOD) and limits of quantification (LOQ) for all pyrethroids were found in the range of 1.6-2.8 and 4.9-8.5 ng/spot, respectively.

**Conclusions:**

The developed method is applicable for separating the mixture of pyrethroids and at the same time, it is also valid for separating their isomers. The method is reproducible, precise and accurate for the quantitative determination of pyrethroids in agricultural and domestic products.

## Background

Chrysanthemum plant (*Chrysanthemum cinerariaefolium*) is known to produce a wide range of natural esters pyrethrins. Pyrethroids are synthetic derivatives of pyrethrins. Historically, pyrethroids were synthesized to maintain the insecticidal activity of pyrethrins while increasing stability to light and resistance time in the environment [1]. Now a days, pyrethroids are widely used to control pests and insects in agriculture, industrial and human habitation [2,3]. They have broad spectrum of insecticidal and pesticidal activity and are less toxic to human and environment [4]. Their use becomes vital as US-EPA (United States Environment Protection Agency) has already banned the use of organophosphate insecticides owing to their toxicity in human.

**Figure 1:**
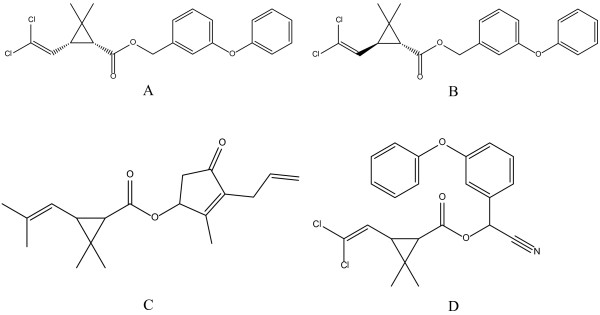
Chemical structures of A = cis-permethrin, B = trans-permethrin, C = esbiothrin, and D = alpha-cypermethrin.

Permethrin, esbiothrin, and alpha-cypermethrin (Figure 1) are commonly used pyrethroids in agriculture and household products. Various formulations of these pyrethroids including foggers, sprays, flea dips, repellent aerosols, liquids, powders, clothing, nets, shampoos, creams, lotions etc. are commercially available. These formulations contain either one or combination of the synthetic pyrethroids. In combination, the toxicity of pyrethroids is enhanced [5]. A lot of health implications are attributed to very high use of pyrethroids. Potential problems to human may include suppressive effects on the immune system [6,7], splenic and lymph node damages and carcinogenesis [8,9]. Pyrethroid insecticides may also show neurotoxicity to mammals [10]. Health effects correlated with inhalation, oral and dermal contact have been well documented by U. S. department of health and human service [11]. Use of pyrethroids in agriculture results in runoff [12] and return flow from irrigated fields results in contamination of streams [13]. Pyrethroids are also extremely toxic to aquatic organism, particularly the fish [14]. Commercially available products show the label claimed pyrethroids content. However, exposure to the pyrethroids exceeding the label directions might adversely affect human health and environment. Hence a careful selection of environment friendly product is required that should contain label claimed pyrethroid amount.

Different methods have been reported for the determination of pyrethroids. Among them, high performance liquid chromatographic (HPLC) methods have been reported for the separation and quantification of permethrin isomers [15-18] and many methods deal with quantitative determination of pyrethroids in various types of biological and environmental samples [19-25]. To the best of our knowledge, thin layer chromatographic method has yet not been reported for the separation and subsequent quantification of pyrethroids in agricultural and domestic products. TLC-densitometry has become a routine analytical technique because of having low operating cost, high sample throughput, and minimum sample clean-up. Moreover, low solvent consumption and shorter analysis time are its advantages over the conventional methods. This paper describes the development of a sensitive and robust TLC-densitometric method for the efficient separation and simultaneous quantification of pyrethroids in agricultural and domestic products. The proposed method is also validated as per the ICH guidelines [26,27].

## Materials and methods

### Standard and reagents

Standard permethrin (mixture of isomers) and esbiothrin were gifted by M/S Reckitt Benckiser (Pakistan) Ltd. Alpha-cypermethrin was obtained from Industrial Analytical Center (IAC), International Center for Chemical and Biological Sciences (ICCBS), University of Karachi. Agricultural and domestic products containing synthetic pyrethroids were procured from The Super Market, Karachi (Pakistan). Methanol, acetone, hexane, dichloromethane, ethylacetate, and formic acid were of analytical grade and purchased from Merck, Germany.

### Purification and characterization of permethrin isomers

Isomeric mixture of permethrin was separated with column chromatography by using a gradient system of pet. ether and ethyl acetate. *Cis*-isomer was eluted at pet. ether:ethyl acetate (92:8) while *trans*-isomer was eluted at pet. ether:ethyl acetate (90:10). Both isomers were characterized through spectroscopic analysis. Moreover, the percent purity of each standard was calculated through GC-MS analysis and found to be > 97%.

### Instrumentation and chromatographic conditions

A CAMAG TLC auto-sampler (Linomat 5) was used for the spotting of all standards and samples. Densitometric scanning was carried out by CAMAG scanner 3. Video densitometry of the chromatosheets was carried out with the help of CAMAG Reprostar 3 and the integrated software of WinCats (Version 1.4.4.6337) was used for the analysis. TLC aluminium sheets precoated with silica gel 60 F-254 (20 cm × 10 cm, MACHEREY-NAGEL, Germany) were used for the application of standards and samples, spotted in the form of bands of width 6 mm with a CAMAG 100 μL syringe.

A constant application rate of 0.1 μL/s was employed and distance between two bands was 9.1 mm. The slit dimension was set to 5 mm × 0.45 mm and 10 mm/s scanning speed was adjusted. The monochromatic bandwidth was kept at 20 nm, each track was scanned thrice and baseline correction was used. Linear ascending development of spotted TLC sheet was carried out in 20 cm × 10 cm twin trough vertical glass chamber (CAMAG) with 10 mL mobile phase (Hexane : dichloromethane : ethyl acetate : formic acid, 8:1.5:0.4:0.1 v/v/v/v) in unsaturation conditions. Chromatographic process was carried out at room temperature (25°C ± 3) and at relative humidity (42% ± 5). Drying of the developed TLC sheet was carried out using air dryer for 5 min. The length of chromatogram run was 8 cm. Densitometric scanning was performed in the reflectance-absorbance mode at corresponding λ_max_ of each standard. The source of radiation utilized was deuterium lamp between 190 to 400 nm. Concentrations of the compounds chromatographed were determined from the intensity of diffusely reflected light. Evaluation was carried out via peak areas using linear regression.

### Sample preparation

Aerosol samples (5 ± 1 mL) were drawn in long glass tube directly from the aerosol container and degassed by sonication for 5 min. 2 μL of degassed aerosol sample was spotted on TLC sheet in triplicate. Liquid samples were spotted after 1:3 dilutions with methanol and 5 μL was spotted in triplicate. However, coil samples were extracted with ethyl acetate. 20 mg of each well grinded coil samples were taken in separate eppendorf tubes and 1.6 mL of ethyl acetate was added into each tube. Coil samples were extracted by sonicating at room temperature for 30 min. In case of mats, samples were extracted by thermomixing at 60°C for 30 min. After extraction process, samples were filtered using 0.44 μm pore size filter paper. Extracts were stored at 4°C until used. 5 μL of each sample extract was spotted in triplicate.

### Calibration curves of standard pyrethroids

The six working standard solutions of each standard pyrethroid were prepared by independently weighing standards and respective volumes were made up with methanol and stored at 4°C until used. 5 μL of each six standard levels (300, 600, 900, 1200, 1500 and 1800 ng/spot) were spotted in triplicate and subjected to chromatographic process, followed by densitometric scanning at λ_max_ 227 nm for both isomers of permethrin, at λ_max_ 228 nm for alpha-cypermethrin and at λ_max_ 238 nm for esbiothrin. This practice was repeated six times to get an average standard calibration curve for each standard pyrethroid in concentration ranges of 300 to 1800 ng/spot. The linearity of standard calibration curves of each standard pyrethroid was verified by residual linearity test and compared with polynomial and power regressions.

### Method validation

#### Precision

Three standard levels (300, 600, 1200 ng/spot) of each pyrethroid were selected for precision study of the developed method. Intra- and inter-day analysis was performed to check the repeatability and reproducibility of the method. For intra-day precision, six replicate analyses of selected standard levels were conducted two times, in a day, while for inter-day precision; six replicate analyses of the same levels were repeated next day. Measurement of % recovery was expressed in terms of relative standard deviation (R.S.D.%). Precision of the whole analytical process was studied by repeating all the steps of mat and coil sample analysis (including sample preparations to final analysis and evaluation).

#### Robustness

By introducing small changes in different parameters of the developed method, the effects on the results were examined. The variables involved changes in scanning wavelength (λ_max_ ± 2 nm), mobile phase composition (hexane: dichloromethane: ethyl acetate: formic acid 8:1.4:0.5:0.1, 8.2:1.3:0.4:0.1, 8.3:1.2:0.4:0.1 v/v/v/v), time from spotting to chromatography (0, 30, 60 min), time from chromatography to scanning (0, 30, 60 min), TLC plate activation (with & without methanol pretreatment), nature of TLC (Al plate, glass plate) and effect of temperature (Room temp. ± 5°C). For each parameter, six chromatograms of each standard level were run. Robustness of the method was accessed at three different standard levels 300, 600 and 1200 ng/spot of each pyrethroid and measurements of % recovery was expressed in terms of relative standard deviation (R.S.D.%). Moreover, robustness testing was applied to real samples, and effects of sample preparation conditions were studied. One-way analysis of variance (ANOVA) was also applied to validate the robustness study.

#### Limit of detection and limit of quantitation

The sensitivity of the method was determined by calculating the limit of detection (LOD) and limit of quantitation (LOQ) of each pyrethroid. LOD & LOQ of each pyrethroid were calculated from corresponding average calibration curve using formula LOD = 3.3 S.D./S, LOQ = 10 S.D./S where, S.D. is the residual standard deviation of regression line or the standard deviation of y-intercept of regression line, and S is the slope of respective calibration curve. The signal to noise ratio 3:1 and 10:1 for LOD and LOQ, respectively were considered. Moreover, both were experimentally confirmed by diluting the known concentration of each pyrethroid standard until the average responses were approximately three or ten times the standard deviation of the responses for six replicate determinations.

#### Specificity

To verify the specificity of the developed method, standards and samples were analyzed simultaneously. The peaks of pyrethroid in samples were confirmed by comparing their *R*_f_ and spectra of the peaks of samples with that of standard pyrethroids. The peak purity of each pyrethroid in samples was assessed by comparing the spectra of standard and samples at three different levels; peak start, peak apex and peak end positions.

#### Recovery studies

Various agricultural and domestic products may have different levels of pyrethroids and thus can possess different matrix effects. To verify the complete extraction of active pyrethroids from samples by extracting solvent, three times pre-analyzed samples were spiked with extra 25, 50 and 75% standard pyrethroid and analyzed by the developed method. The practice was performed for mat and coil samples to assure the complete extraction of esbiothrin. However, aerosol and liquid samples were not gone through this practice as they were analyzed without the extraction process. The analysis of spiked samples was repeated six times.

## Results and discussion

### Extraction methodology and extraction efficiency

**Figure 2:**
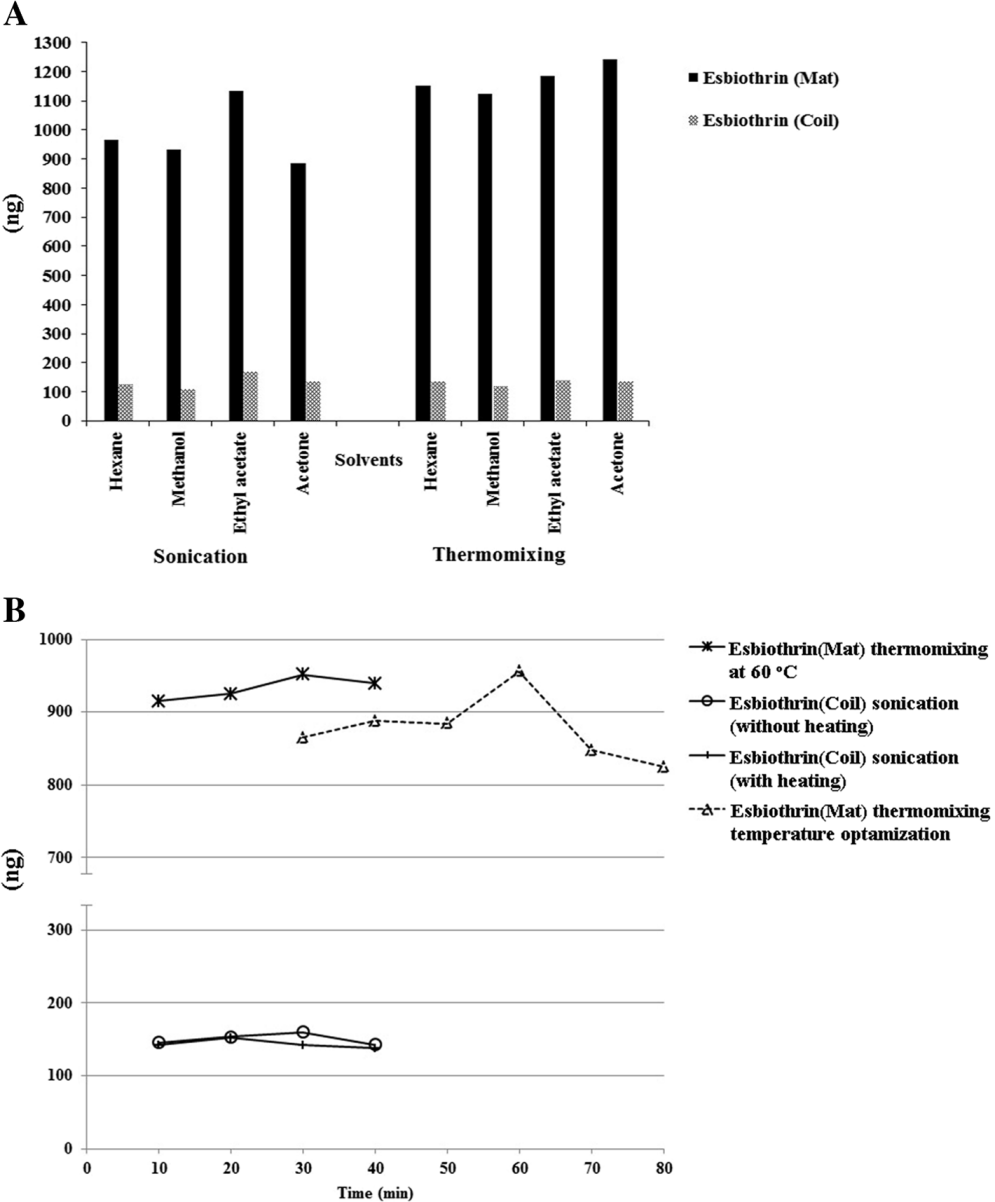
**A = Optimization of extraction methodology and B = Time optimization for sonication with and without heating.** X-axis represents the temp. (°C) only for dotted (……) trend line.

Various strategies have been reported in the literature for the extraction of pyrethroids from their formulations [15,23,28,29]. Our aim was to develop a simple, cost effective and efficient extraction methodology. This study is unique in the sense that aerosol and liquid samples were directly analyzed without going through extraction process. However, mat and coil samples went through the process of esbiothrin extraction. Four different solvent systems including hexane, methanol, ethyl acetate and acetone, treated with thermomixer and sonication at various temperature and time durations, were applied to evaluate the extraction efficiency of esbiothrin from its corresponding samples. Ethyl acetate proved to be the best extracting solvent for esbiothrin from coil based samples. However, in comparison between sonication and thermomixing, the former one was found to be more efficient. Esbiothrin was best extracted by sonication for 30 min without heating. In case of mat samples having esbiothrin as an active ingredient, acetone proved to be the best extracting solvent by thermomixing at 60°C for 30 min. (Figure 2).

### Development of the optimum mobile phase

**Figure 3:**
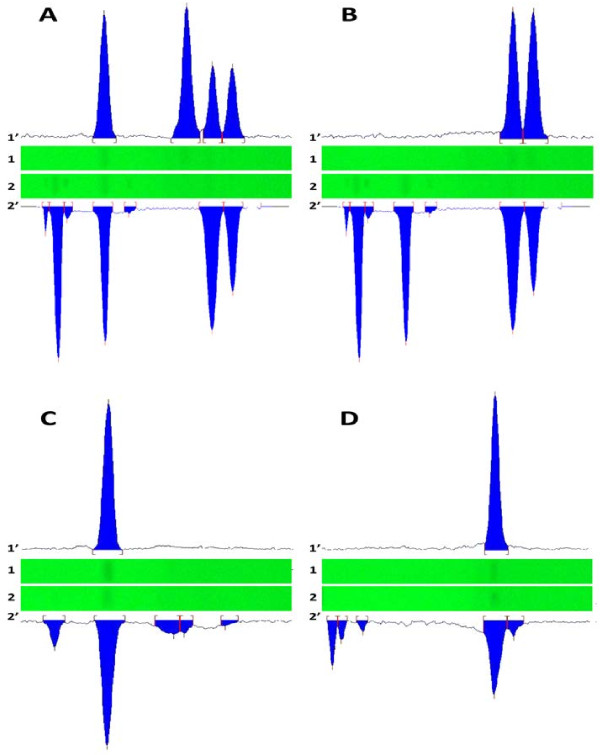
**Videodensitometries and UV chromatograms of mixture of all standards (A), mixture of *cis- *and *trans*-permethrin (B), esbiothrin (C) and alpha-cypermethrin (D).** 1 & 2 are chromatosheets of standards and samples respectively and 1′ & 2′ are corresponding chromatograms of standards and samples respectively.

We were interested to develop a common mobile phase for all pyrethroids under study. A mobile phase that should hold the uniqueness of performing the dual function of well separating the pyrethroids and giving good resolution of geometrical isomers of permethrin. Samples and standards were simultaneously spotted and developed in different solvent systems. Various combinations of hexane were tried with diethyl ether. These combinations gave poor resolution of the isomers of permethrin. A gradient system consisting of hexane, diethyl ether and ethyl acetate gave a reasonable separation of the isomers, however *R*_f_ values were little perplexing. Replacement of diethyl ether by dichloromethane resulted in better separation. Finally, the mobile phase consisting of hexane: dichloromethane: ethyl acetate: formic acid in a ratio of (8:1.5:0.4:0.1 v/v/v/v), provided sharp and symmetrical peaks with improved spots characteristics. The pyrethroids peaks were well separated under unsaturation conditions at room temperature having *R*_f_ values of 0.65, 0.6, 0.53 and 0.31 for *cis*-permethrin, *trans*-permethrin, alpha-cypermethrin and esbiothrin, respectively (Figure 3).

### Standard calibration curves

Six standard levels of each pyrethroid were spotted simultaneously on TLC sheets in triplicate. Densitometric scanning was recorded at λ_max_ 227 nm for both isomers of permethrin, at λ_max_ 228 nm for alpha-cypermethrin and at λ_max_ 238 nm for esbiothrin. This practice was repeated six times for each pyrethroid to get its average calibration curve. Linearity was found to be *r* = 0.996 ± 0.0015, 0.993 ± 0.0029, 0.991 ± 0.0016, and 0.996 ± 0.0032 for the calibration curves of *cis*-permethrin, *trans*-permethrin, esbiothrin, and alpha-cypermethrin, respectively, in a concentration range of 300 to 1800 ng/spot. When linear regression was compared with polynomial and power regressions, R^2^ values for polynomial and power regressions were little high than that of linear regression lines (Figure 2, Additional file 1). However, in case of linear regression, linearity values for all pyrethroids were in acceptable range (> 0.99). The regression data is shown in Table 1. In residual linearity test of all pyrethroids, the random distribution of residuals against respective standard levels reported a linear model of standard calibration curves of each pyrethroid (Figure 1, Additional file 1).

**Table 1:** **Linear regression data for calibration curves of standards (*n *= 6) via peak areas**

**Standard**	**Linearity range [ng/spot]**	**Regression equation**	***r*± SD**	**LOD [ng/spot]**	**LOQ [ng/spot]**
*Cis*-permethrin	300-1800	*y* = 6.56*X* + 1649.4	0.996 ± 0.0015	1.6	4.9
*Trans*-permethrin	300-1800	*y* = 5.84*X* + 1876.2	0.993 ± 0.0029	2.4	7.4
Esbiothrin	300-1800	*y* = 4.33*X* + 1283.3	0.991 ± 0.0016	2.8	8.5
Alphacypermethrin	300-1800	*y* = 5.3*X* + 1295.6	0.996 ± 0.0032	1.9	5.7

### Validation of the method

#### Precision

The repeatability of the method was expressed in percent relative standard deviation between two analyses in terms of % recovery of three selected standard levels of each pyrethroid and was found to be ≤ 3.07 for all three levels of each pyrethroid ( Additional file 1: Table S1). Similarly, there was no significant difference of intra- and inter-day analysis and R. S. D.% in all cases was < 1.5. Statistical data is shown in Table 2. However, in case of precision of whole analytical process, R. S. D. between two yields of esbiothrin in repeated analysis of mat sample was 0.92% and in case of coil sample, R. S. D. between two yields of esbiothrin in repeated analysis was 2.59%.

**Table 2:** **Intra- and inter-day analysis (*n *= 6)**

**Standard**	**Intra-day precision**				**Inter-day precision**			
	**Amount fractioned (ng)**	**% recovery**^**a)**^		**% RSD**	**Amount fractioned (ng)**	**% recovery**^**a)**^		**% RSD**
		**1**^**st**^**time**	**2**^**nd**^**time**			**Day1**	**Day2**	
*Cis*-permethrin	300	105.7	105.3	0.28	300	105	104	0.766
	600	97	96.74	0.2	600	97	97	0.0
	1200	99.73	99.57	0.1	1200	99.7	99.6	0.1
*Trans*-permethrin	300	103.5	103.4	0.097	300	103	102	0.78
	600	98.33	97.44	0.61	600	98.3	98.2	0.1
	1200	100.2	100.4	0.199	1200	100	100	0.0
Esbiothrin	300	102.5	102.1	0.29	300	102	102	0.0
	600	96.88	96.6	0.2	600	96.8	95.3	1.15
	1200	99.85	99.17	0.5	1200	99.8	99.4	0.3
Alpha-cypermethrin	300	97.87	99.81	1.42	300	97.9	97.7	0.1
	600	100.5	100	0.299	600	100	101	0.697
	1200	99.9	99.9	0.0	1200	99.9	99.4	0.3

^a)^Values are mean of n = 6.

#### Robustness

The standard deviation of % yield of three standard levels in repeated trials was calculated for each parameter; R. S. D.% was < 2.4 for all parameters in case of all pyrethroids ( Additional file 1: Table S2). In case of sample preparation conditions, R. S. D. was 1%, for yields of esbiothrin in mat samples during variation in thermomixing temperature (Optimum temp. ± 5°C), and in case of variation in sonication time (Optimum time ± 5 min.), R. S. D. was 2.19% for the yields of esbiothrin in coil sample. The robustness study was validated by applying one-way ANOVA on the % recovery of four compounds. The results of this statistical evaluation are highlighted in, Additional file 1: Table S3. With 95% confidence, it can be concluded that there is no significant effect on % recovery of pyrethroid by small variation in robustness factors.

#### LOD and LOQ

The LODs with signal/noise ratio of 3:1 were observed to be 1.6, 2.4, 2.8, and 1.9 ng/spot for *cis-*permethrin, *trans-*permethrin, esbiothrin, and alpha-cypermethrin respectively, while LOQs with signal/noise ratio 10:1 were found to be 4.9, 7.4, 8.5, and 5.7, ng/spot respectively, summarized in Table 1.

#### Specificity

The peak purities of standard pyrethroids and that of samples were evaluated by comparing their respective spectra at peak start, peak apex and peak end positions. Good correlations, *r* (start, middle) = 0.9999 and *r* (middle, end) = 0.9998, *r* (start, middle) = 0.9997 and *r* (middle, end) = 0.9995, *r* (start, middle) = 0.9998 and *r* (middle, end) = 0.9991, and *r* (start, middle) = 0.9995 and *r* (middle, end) = 0.9997 were observed by comparing the spectra of *cis*-permethrin, *trans*-permethrin, esbiothrin, and alpha-cypermethrin standard and corresponding peaks in samples, respectively. The overlay spectra of respective standards and corresponding peaks in the samples (Figure 3, Additional file 1) indicated that there were no other peaks at the retention factors of each pyrethroid. λ_max_ of each pyrethroid could also be estimated from the corresponding overlay spectra.

#### Analysis of agricultural and domestic products

Twenty one samples of pyrethroid insecticides including 6 aerosols, 5 liquids, 2 coils, 7 mats and an oil spray samples were investigated for the quantitative analysis of active ingredients. All samples that claimed permethrin as active ingredient were found to possess two isomers of permethrin (*cis* and *trans*). Peaks of *trans* and *cis-*permethrin were observed at *R*_f_ 0.6 and 0.65, respectively in all samples (Figure 3). Peaks of *trans* and *cis* isomers of permethrin were sharply separated while peaks of interfering compounds in all samples were distant away from the analytes peaks. Sum of the isomeric yields was in the agreement with label claimed permethrin contents. Geometrical isomers were not found in case of other pyrethroids. All agricultural and domestic samples were containing the only label claimed ingredients. Results of all analyzed samples of various agricultural and domestic products are summarized in Table 3.

**Table 3:** Quantitative analysis of pyrethroid insecticides in various agricultural and domestic products

**Product name**	**State**	**Usage**	**Labeled active ingredient**	**Label claim**	**Experimental yield (% w/w)**	**S.D**^**a)**^
ARS	Aerosol	Domestic	Permethrin	0.3%	*cis* = 0.078	0.03
					*trans* = 0.21	
					Sum = 0.288	
Baygon	Aerosol	Domestic	Permethrin & Esbiothrin	0.1%	*cis* = 0.032	0.67
				0.1%	*trans* = 0.07	0.0015
					Sum = 0.102	
					0.089	
Mortein PG	Aerosol	Domestic	Permethrin & Esbiothrin	0.3%	*cis* = 0.03	0.044
				0.126%	*trans* = 0.25	0.002
					Sum = 0.28	
					0.13	
Power Plus	Aerosol	Domestic	Permethrin	0.3%	*cis =* 0.08	
					*trans* = 0.22	
					Sum = 0.3	
Kingtox	Aerosol	Domestic	Permethrin & Esbiothrin	0.3%	*cis* = 0.1	0.036
				0.27%	*trans* = 0.24	0.002
					Sum = 0.34	
					0.24	
Mirage	Aerosol	Domestic	Permethrin	0.3%	*cis* = 0.07	
					*trans* = 0.3	
					Sum = 0.37	
Permax	Liquid	Agricultural& domestic	Permethrin	25 g/L	*cis* = 9.3 g/L	
					*trans* = 15.5 g/L	
					Sum = 24.8 g/L	
Coopex	Oil Spray	Agricultural& domestic	Permethrin	0.05%	*cis* = 0.01	
					*trans* = 0.035	
					Sum = 0.045	
Fendona	Liquid	Agricultural& domestic	Alpha-cypermethrin	100 g/L	103.18 g/L	1.34
Guardian +	Liquid	Agricultural& domestic	Alpha-cypermethrin	100 g/L	99.77 g/L	0.95
Console	Liquid	Agricultural& domestic	Alpha-cypermethrin	100 g/L	102.64 g/L	1.2
Alpha-Hit	Liquid	Agricultural& domestic	Alpha-cypermethrin	100 g/L	93.78 g/L	0.61
Baygon	Coil	Domestic	Esbiothrin	0.15%	0.12%	0.01
Finis	Coil	Domestic	Esbiothrin	0.15%	0.13%	0.01
Power plus	Mat	Domestic	Esbiothrin	40 mg/mat	40.89 mg/mat	2.36
Finis	Mat	Domestic	Esbiothrin	40 mg/mat	38.02 mg/mat	1.24
Metro	Mat	Domestic	Esbiothrin	40 mg/mat	36.58 mg/mat	0.69
Mortein PG	Mat	Domestic	Esbiothrin	NA	1.51%	0.03
Mortein NG	Mat	Domestic	Esbiothrin	NA	1.61%	0.05
King	Mat	Domestic	Esbiothrin	NA	2.05%	0.06
Tiger	Mat	Domestic	Esbiothrin	NA	1.95%	0.1

a) Mean of standard deviations of replicate measurements, NA = not available, NF = not found.

## Conclusions

In conclusion, the proposed TLC-densitometric method for the separation and subsequent quantitative analysis of pyrethroid insecticides in broad range of agricultural and domestic products including aerosols, liquids, coils, mats and oil is found to be precise, repeatable, accurate and robust. Proposed method is very simple, exhibits low cost sample analysis and applicable for analytical and quality control assays of pyrethroid insecticides in domestic and agricultural insect controlling formulations. Moreover, the present study is a multipurpose report describing a thin layer chromatographic method for the separation of not only the pyrethroids from each other but it also serves as a tool for the separation of their geometrical isomers and simultaneous quantification of pyrethroids and their isomers. This study can also be useful for the health, environmental and industrial R & D authorities in the world.

## Competing interests

Authors declare that they have no competing interests.

## Authors’ contributions

SGM: Supervised the whole study and participated in method optimization. MS: Participated in experimental designing and involved in performing experimental and manuscript preparation. DK: Participated in bench work. MNH: Involved in useful discussions and participated in manuscript preparation. All authors read and approved the final manuscript.

## Supplementary Material

Additional file 1 **Table S1.** Recovery studies (n = 6). **Table S2.** Robustness testing (*n* = 6). **Table S3.** Analysis of variance (ANOVA) of robustness factors. Click here for file
