# Supplementary material for: Validated TLC-densitometry method for the simultaneous analysis of pyrethroid insecticides in agricultural and domestic products
Source: Chem Cent J. 2012 Aug 31;6:93. doi: 10.1186/1752-153X-6-93 (PMC3496573; doi:10.1186/1752-153X-6-93)
Supplement: Additional file 1 — Table S1. Recovery studies (n = 6). Table S2. Robustness testing (n = 6). Table S3. Analysis of variance (ANOVA) of robustness factors. [file 1752-153X-6-93-S1.doc]

**Additional File 1**

**Table S1 Recovery studies (*n* = 6)**

| Standard | Type of sample | Standard spiked [%] | Theoretical [ng/spot] | Experimental [ng/spot] | Recovery [%] | % RSD |
| --- | --- | --- | --- | --- | --- | --- |
| Esbiothrin | Mats | 25  50  75 | 692.5  831  969 | 682.5  828  984 | 98.56  99.65  101.5 | 1.26  0.73  0.36 |
|  | Coils | 25  50  75 | 148.8  195.5  242.2 | 148.5  201.9  250 | 99.75  102.8  103.2 | 3.07  1.09  1.52 |

**Table S2** Robustness testing (*n* = 6).

| Standard | Parameter | SD^a)^ of % yield | % RSD ^a)^ |
| --- | --- | --- | --- |
| *Cis*-permethrin | Effect of wavelength  Mobile phase composition  Time from spotting to chromatography  Time from chromatography to scanning  Effect of TLC plate activation  Nature of TLC plate  Effect of temperature | 0.23  1.98  1.32  1.89  1.15  1.11  0.87 | 0.22  2.09  1.29  1.86  1.14  1.06  0.89 |
| *Trans*-permethrin | Effect of wavelength  Mobile phase composition  Time from spotting to chromatography  Time from chromatography to scanning  Effect of TLC plate activation  Nature of TLC plate  Effect of temperature | 0.56  1.87  1.8  1.42  2.17  2.46  1.08 | 0.55  1.84  1.81  1.37  2.12  2.39  1.1 |
| Esbiothrin | Effect of wavelength  Mobile phase composition  Time from spotting to chromatography  Time from chromatography to scanning  Effect of TLC plate activation  Nature of TLC plate  Effect of temperature | 0.21  1.52  1.75  1.83  1.58  1.36  0.47 | 0.21  1.58  1.73  1.81  1.57  1.36  0.48 |
| Alpha-cypermethrin | Effect of wavelength  Mobile phase composition  Time from spotting to chromatography  Time from chromatography to scanning  Effect of TLC plate activation  Nature of TLC plate  Effect of temperature | 0.26  1.08  0.75  1.09  0.89  0.92  0.99 | 0.27  1.08  0.75  1.07  0.89  0.92  1.01 |

^a)^Average of three concentrations 300, 600 and 1200 ng per spot

**Table S3** Analysis of variance (ANOVA) of robustness factors.

| Parameters | Mean % Recovery ± S.D. | F-calculated | F-table (ν_1_, ν_2_) |
| --- | --- | --- | --- |
| Scanning Wavelength | 100.968  ± 3.823 | 0.3747 | 3.3 (2,33) |
| Mobile Phase | 101.040  ± 4.197 | 0.3761 | 2.8 (3,44) |
| Time form Spotting to Chromatography | 101.128  ± 3.016 | 0.0744 | 3.3 (2,33) |
| Time from Chromatography to Scanning | 101.002  ± 3.087 | 0.2333 | 3.3 (2,33) |
| Temperature | 98.369  ± 1.306 | 2.1326 | 3.3 (2,33) |

P=0.05, 95% confidence limit.


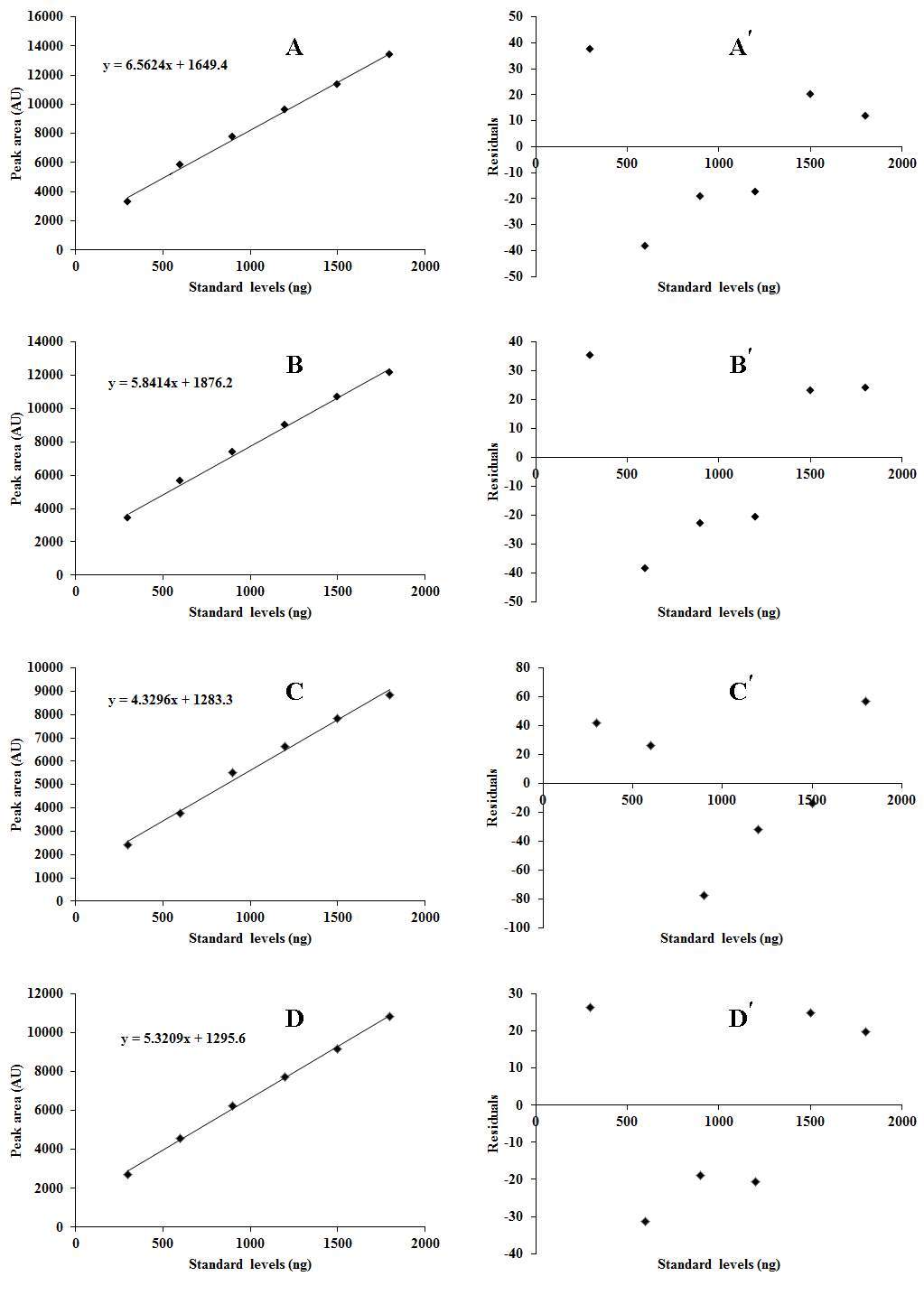


**Figure 1.** Standard calibration curves. A = *cis*-permethrin, B = *trans*-permethrin, C = esbiothrin, D = alpha-cypermethrin, and A′, B′, C′, & D′ are residual linearity tests, respectively.


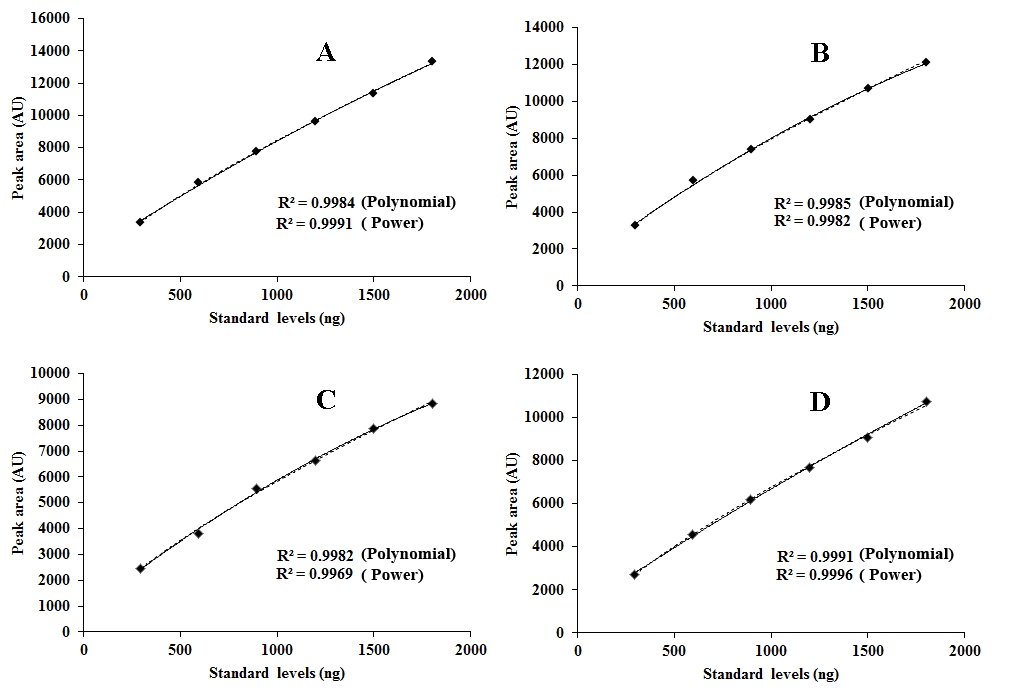


**Figure 2.** Polynomial (solid line) and power (dotted line) regression lines, A = *cis*-permethrin, B = *trans*-permethrin, C = esbiothrin and D = alpha-cypermethrin.


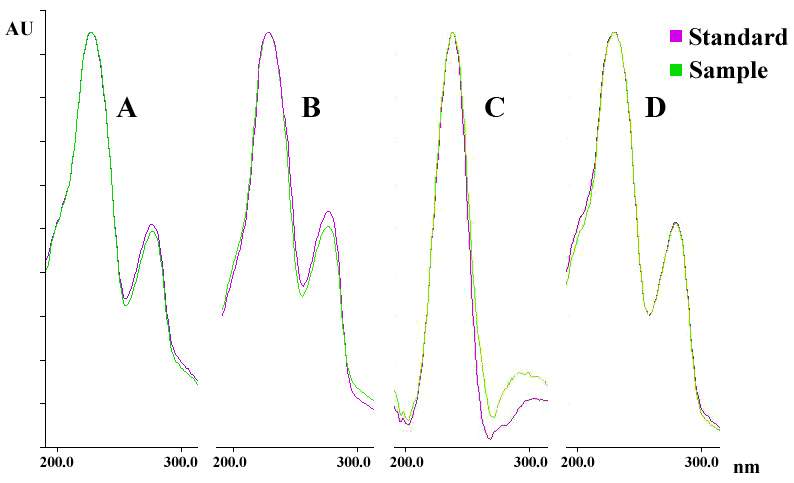


**Figure 3.** Overlay spectra of standard and sample in absorption mode in the UV range, taken on the CAMAG TLC scanner 3. A = *cis*-permethrin, B = *trans*-permethrin, C = esbiothrin, D = alpha-cypermethrin.
